# Pediatric Tape: Accuracy and Medication Delivery in the National Park Service

**DOI:** 10.5811/westjem.2015.6.25618

**Published:** 2015-10-20

**Authors:** Danielle D. Campagne, Megann Young, Jedediah Wheeler, Geoff Stroh

**Affiliations:** University of California, San Francisco – Fresno Medical Education Program, Department of Emergency Medicine, Fresno, California

## Abstract

**Introduction:**

The objective is to evaluate the accuracy of medication dosing and the time to medication administration in the prehospital setting using a novel length-based pediatric emergency resuscitation tape.

**Methods:**

This study was a two-period, two-treatment crossover trial using simulated pediatric patients in the prehospital setting. Each participant was presented with two emergent scenarios; participants were randomized to which case they encountered first, and to which case used the National Park Service (NPS) emergency medical services (EMS) length-based pediatric emergency resuscitation tape. In the control (without tape) case, providers used standard methods to determine medication dosing (e.g. asking parents to estimate the patient’s weight); in the intervention (with tape) case, they used the NPS EMS length-based pediatric emergency resuscitation tape. Each scenario required dosing two medications (Case 1 [febrile seizure] required midazolam and acetaminophen; Case 2 [anaphylactic reaction] required epinephrine and diphenhydramine). Twenty NPS EMS providers, trained at the Parkmedic/Advanced Emergency Medical Technician level, served as study participants.

**Results:**

The only medication errors that occurred were in the control (no tape) group (without tape: 5 vs. with tape: 0, p=0.024). Time to determination of medication dose was significantly shorter in the intervention (with tape) group than the control (without tape) group, for three of the four medications used. In case 1, time to both midazolam and acetaminophen was significantly faster in the intervention (with tape) group (midazolam: 8.3 vs. 28.9 seconds, p=0.005; acetaminophen: 28.6 seconds vs. 50.6 seconds, p=0.036). In case 2, time to epinephrine did not differ (23.3 seconds vs. 22.9 seconds, p=0.96), while time to diphenhydramine was significantly shorter in the intervention (with tape) group (13 seconds vs. 37.5 seconds, p<0.05).

**Conclusion:**

Use of a length-based pediatric emergency resuscitation tape in the prehospital setting was associated with significantly fewer dosing errors and faster time-to-medication administration in simulated pediatric emergencies. Further research in a clinical field setting to prospectively confirm these findings is needed.

## INTRODUCTION

Since its inception in the 1980s, the pediatric emergency tape (commonly called the Broselow® Tape)^1^ has been used as a method to quickly and safely determine medication doses for pediatric patients whose weight is unknown. There has been considerable research regarding the accuracy of medication dosing using the Broselow® Tape.^2^–^12^ Relatively little research has investigated the use of a length-based tape in the prehospital setting;^13^–^15^ however, these studies have shown that prehospital providers can accurately use a length-based pediatric emergency resuscitation tape for medication dosing.^13^–^15^

Based on the concept of the Broselow® Tape and with the aid of a grant from the National Park Foundation, a novel length-based pediatric emergency resuscitation tape specifically tailored to the National Park Service (NPS) Emergency Medical Services advanced life support (ALS) scope of practice (SOP) was developed for use in the NPS. This SOP encompasses 32 medications, including cardiac drugs, narcotics, antibiotics and many others. The tape was also designed to be more durable in the austere environments encountered on a routine basis in the National Parks.

The austere and remote environments of the National Parks often lead to long transport times and extended patient contacts; for this reason, there is an expanded SOP for ALS providers in the NPS. These ALS providers are referred to as Parkmedics^16^ and have a SOP between that of an advanced emergency medical technician and paramedic.

The aim of this study was to evaluate the accuracy of medication dosing as well as time to administration of medication dose by NPS Parkmedics using this novel length-based tape in simulated prehospital pediatric scenarios.

## METHODS

### Setting/Population

Every two years, a new group of Parkmedics is trained in Fresno, California, under the auspices of the UCSF-Fresno Emergency Medicine Department and Community Regional Medical Center. During their training course, along with standard training on medication calculation and administration, Parkmedic students were introduced to the novel length-based pediatric emergency resuscitation tape and received training regarding its use, including head-to-toe placement and locating corresponding medication doses. Each Parkmedic was invited to participate in the study at the end of his/her course; participation was completely voluntary and independent of their training program.

### Study Design

The study was designed as a two-period, two-treatment crossover trial. Each Parkmedic participated in two simulated emergent pediatric scenarios. The 20 participants were randomly assigned to one of four groups by drawing cards. Half were assigned to case one (febrile seizure) first and case two (anaphylaxis) second. The others had the opposite assignments. Each of these two groups was again divided, with half assigned to control (without tape) first and intervention (with tape) second. The others again had the opposite assignments. The study design flowchart is presented in the accompanying figure. As the researchers were actively involved in the acquisition of data, there was no blinding as to the assignments.

### Institutional Review Board Approval

This study received approval from the Community Medical Centers/UCSF Fresno Institutional Review Board.

### Intervention/Treatments

When given an intervention (with tape) case, the Parkmedics used the length-based tape to determine the doses of the appropriate medications to be given in the scenario. During the control (without tape) scenarios, the Parkmedics used standard methods of determining the medication dose. These methods include, but are not limited to, asking the simulated parent for the weight of the patient; estimating the patient’s weight; looking up the weight or age-based medication dose in a protocol or other reference; or calling the simulated base hospital for dosing. If estimating the patient’s weight, the Parkmedic would also need to calculate the appropriate weight-based dose for each medication.

### Outcome Measures

This study had two primary outcome measures: accuracy of drug dose using the length-based tape compared with the accuracy of the dose using standard methods of dose acquisition; and the time it took to determine the dose of each drug using the length-based tape compared with time to determination of dosing using the standard methods of dose acquisition. We defined a dosing error for each drug as being a >25% deviation from the correct weight-based dose for each drug; this is consistent with other studies of medication dosing errors in pediatric patients, where acceptable error percentages range from 10–25%.^17^–^19^

### Study Protocol

Each Parkmedic participated in two simulated emergent prehospital pediatric scenarios. In each scenario, it was appropriate to give two medications. Scenario 1 was a 9 pound/4kg infant with febrile seizure requiring both midazolam and acetaminophen. Scenario 2 was a 22 pound/10kg toddler with anaphylaxis requiring both epinephrine and diphenhydramine. Mannequins were used as simulated patients and were selected for appropriate size (length) for use with the tape. The subjects were oriented to the scenarios including simulated access to base hospital; use of protocols; simulated access to the patients’ parents during all scenarios; and access to the length-based tape during the intervention scenarios. They were asked to voice out loud the moment they decided a medication should be given and similarly voice out loud the dose to be given as soon as the dose was determined. The scenarios began with each proctor reading a basic description of the clinical situation. History, vital signs, physical exam findings, and response to treatment were all voiced by the proctors as appropriate throughout the scenario. The timer for each medication was started at the moment the decision to give the appropriate medication was indicated and stopped when the dose was decided. The time and dose were recorded for each medication in each case.

### Analytical Methods

We used Microsoft Excel 2007 (Microsoft Inc., Redmond, WA) for data collection and statistical analysis. Fisher’s exact test was used to compare the drug dose data, and we used the t-test for independent samples to interpret the time data.

## RESULTS

### Dose Error

Twenty Parkmedics participated in a total of 40 scenarios and delivered medication 78 times. (One provider did not give acetaminophen during their control case, and another provider did not give diphenhydramine during their control case; these omissions were not included in the statistical analysis as dosing errors.) See Table 2a and 2b for all results. We defined a medication dose error as a greater than 25% deviation from the appropriate weight-based dose, with the exception of epinephrine. For true weight-based dosing, the correct epinephrine dose for a 10kg patient would be 0.1mg; however, on the tape the correct dose is 0.3mg, so both doses were accepted as correct. (See dosing in NPS protocol in discussion section.) Acceptable dose ranges for each medication are listed in Table 1.

Acetaminophen doses were 45mg (11.25mg/kg), 60mg (15mg/kg), 61mg (15.25mg/kg) and a single outlier of 135mg (33.75mg/kg) that occurred in the control group and was considered the only error for acetaminophen administration. Midazolam doses were 0.4mg (0.1mg/kg) and 0.5mg (0.125mg/kg) with a single outlier of 1.5mg (0.375mg/kg) that occurred in the control group and was considered the only error for midazolam administration. Diphenhydramine doses were 10mg (1mg/kg) and 12mg (1.2mg/kg) for all but two cases; the two errors were doses of 22mg (2.2mg/kg) and 1mg (0.1mg/kg), both in the control group, which were considered the only errors for diphenhydramine administration. Epinephrine was given in 0.1mg (0.01mg/kg), 0.2mg (0.02mg/kg) and 0.3mg (0.03mg/kg) doses with one outlier of 1mg (0.1mg/kg), which was in the control group and considered the only error for epinephrine. Overall, there were five errors in the control group and no errors in the intervention group (p=0.024) (Table 3a).

### Time to Determination of Dose

The mean time to determination of acetaminophen dose in the intervention (with tape) group was shorter at 28.6s (12–90s) than the 50.6s (24–90s) in the control group (p=0.036). The mean time to determination of midazolam dosing in the intervention (with tape) group was shorter at 8.6s (5–18s) than the 27s (10–58s) in the control group (p=0.005). There was no difference between the groups in time required to determine the dose for epinephrine (intervention group: 23.3s [1–50s] vs. control group: 22.9s [7–56s], p=0.96). The mean time to determination of diphenhydramine dose was shorter in the intervention group (13s [2–28s]) than in the control group (37.6s [19–65s]) (p=0.0005) (Table 3b).

## DISCUSSION AND LIMITATIONS

### Weight Based Dosing and Acceptable Error

As stated above, for the drugs in our study we defined a medication dose error as a greater than 25% deviation from the appropriate weight-based dose. We consulted with our hospital pharmacist; performed a literature search using the search words “pediatric drug error range,” “pediatric drug error percent,” “acceptable pediatric drug dose range,” and “pediatric drug dosing;” and calculated percent error from the Broselow® Tape, all in an attempt to determine a literature-based acceptable error for pediatric medication dosing. Using the numbers for epinephrine and lorazepam from the Broselow® Tape at the Purple (10–12kg) zone and accepting the known variance from child to child with respect to the weight/length proportion, a potential 20% variance in dosing is within the tolerance recommended by the tape. We concur with this range and although there is little literature to support the safety of tolerating higher dosing errors, anecdotally and from previous high-dose epinephrine use–common and pediatric advanced life support recommended until relatively recently–we believe that a 25% range for dosing error is reasonably safe. This is for a single dose administration and cumulative doses would, of course, carry additional risks. Lastly, we emphasize the importance of error reduction in all medication dosing, but simultaneously recognize that with lifesaving therapies, some overdosing is likely preferable to underdosing, and that, conversely, underdosing would be preferable with so-called comfort medications.

This study adds to the body of literature demonstrating that a pediatric length-based tape can reduce medication dosing errors, decrease time to administration and potentially improve overall safety in pediatric resuscitation. Additionally, this study shows that NPS Parkmedics can safely use this tool in the prehospital setting.

There were no medication dosing errors in the intervention (tape) group; all five errors occurred in the control (no tape) group. This is a statistically significant difference. The errors were spread across the different variables, with two errors in case 1 and three errors in case 2, and at least one error for each drug. They were also distributed across periods of the study, with three errors occurring during the subjects’ first scenario and two errors during the second scenario. This suggests that learning bias was minimized. The clinical relevance of the dosing errors is somewhat less obvious, however; it is not clear that the medication errors made would have led to adverse clinical consequences. Perhaps the most clinically relevant errors were the 10× overdosing of epinephrine (this dose is too high for even an adult patient) and the 10× underdosing of Benadryl. (This low a dose is unlikely to have its intended clinical effect.) A dose of epinephrine this high would almost certainly lead to significant tachycardia and hypertension in a 10kg child, if not to more serious unintended effects. There were no such errors in the group using the pediatric resuscitation tape. Underdosing both epinephrine and versed are also likely to be clinically significant, since at low doses they are unlikely to achieve their intended therapeutic effect. We did not observe significant underdosing of these medications in this study; but the overall increased accuracy with the pediatric emergency resuscitation tape suggests that this type of error would be minimized as well with widespread use of such a resuscitation tape.

In evaluating time-to-medication dosing, there was a significant difference for all of the drugs except epinephrine. There are several factors that may explain this. First, during the course it was emphasized that epinephrine is a truly lifesaving medication and the students were expected to memorize epinephrine dosing for all patients; the same was not true for the other medications used in this study. In addition, the protocols for the NPS are written such that the same dose of epinephrine is acceptable across a wide range of patient weights; the rationale behind this is that giving a higher dose of a lifesaving medication is likely to be better than a delay to administration due to difficulty in calculating the correct dose. This combination of factors probably explains the uniformity of time-to-epinephrine dosing. For the other medications, however, it appears that use of the length-based tape resulted in faster determination of medication dosing. Rapid medication dosing is most important in critically ill patients and for potentially lifesaving medications, which were emphasized in this study. While there is no guarantee that faster determination of drug dosing would result in faster drug administration, these two factors are likely positively correlated. If it is true that faster determination of drug dosing leads to faster drug administration, this would be clinically significant for some drugs such as epinephrine and midazolam.

This study was subject to several limitations. First, the study has a small sample size. All students in the Parkmedic course were invited to participate, and 20 of them did (out of 24 students in the class). Participation was not required, and participation in the study had no bearing on class standing. Second, the study used a crossover design. Learning bias is always a potential problem in crossover studies (that is, performance in period 2 scenarios is improved by things learned during period 1 participation). We attempted to minimize learning bias in the study design, with randomization of both the case encountered first, as well as the intervention (with tape) versus control assignments. In this design, any learning carried over into the second period would affect both the control and intervention groups equally. In addition, the two cases differed in patient age and clinical presentation, thus minimizing the amount of learning that could be carried over into the second period. Third, the study was carried out using simulated scenarios and mannequins instead of real patients or live simulated patients. There is some loss of fidelity in any simulated scenario, but this should have affected all participants and both cases equally, thus minimizing any bias.

## CONCLUSION

This study demonstrates that NPS Parkmedics can use a novel length-based pediatric emergency resuscitation tape, which was associated with a significantly lower number of dosing errors and faster time to determination of medication dosing in simulated pediatric emergencies. Further research is needed to investigate its impact in a clinical setting.

## Figures and Tables

**Figure:**
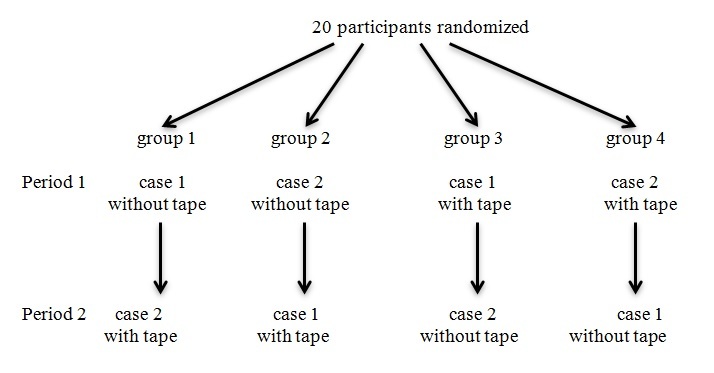
Design of the two-period, two-treatment crossover trial; “intervention” denoted use of the length-based tape.

**Table 1:** Accepted dosage ranges in study examining use of pediatric emergency resuscitation tape by National Park EMTs.

Drug	Accepted dose range (mg)
Midazolam	0.3–0.6
Acetaminophen	45–75
Epinephrine	0.1–0.3
Diphenhydramine	7.5–12.5

*EMT,* emergency medical technician

**Table 2a:** Case one - febrile seizure. Dose and time for each medication in each group.

Treatment	Medication	Dose (mg)	Time (s)	Medication	Dose (mg)	Time (s)
Intervention	Acetaminophen	60	15	Midazolam	0.5	5
Intervention	Acetaminophen	45	16	Midazolam	0.5	10
Intervention	Acetaminophen	60	12	Midazolam	0.5	5
Intervention	Acetaminophen	60	25	Midazolam	0.5	18
Intervention	Acetaminophen	60	90	Midazolam	0.4	14
Intervention	Acetaminophen	60	23	Midazolam	0.5	5
Intervention	Acetaminophen	45	16	Midazolam	0.5	8
Intervention	Acetaminophen	45	22	Midazolam	0.5	6
Intervention	Acetaminophen	60	27	Midazolam	0.5	7
Intervention	Acetaminophen	60	40	Midazolam	0.5	5
Control	Acetaminophen	60	90	Midazolam	0.4	10
Control	Acetaminophen	60	40	Midazolam	0.4	16
Control	Acetaminophen	60	55	Midazolam	0.4	58
Control	Acetaminophen	60	63	Midazolam	0.4	30
Control	Acetaminophen	60	44	Midazolam	0.4	16
Control	Acetaminophen	135	49	Midazolam	1.5	40
Control	Acetaminophen	60	52	Midazolam	0.4	27
Control	Acetaminophen	60	24	Midazolam	0.4	24
Control	Acetaminophen	61	38	Midazolam	0.4	14
Control	Acetaminophen	not given	not given	Midazolam	0.4	35

**Table 2b:** Case two - anaphylaxis. Dose and time for each medication in each group.

Treatment	Medication	Dose (mg)	Time (s)	Medication	Dose (mg)	Time (s)
Intervention	Epinephrine	0.3	27	Diphenhydramine	10	10
Intervention	Epinephrine	0.3	39	Diphenhydramine	10	4
Intervention	Epinephrine	0.1	1	Diphenhydramine	10	20
Intervention	Epinephrine	0.3	15	Diphenhydramine	10	12
Intervention	Epinephrine	0.3	5	Diphenhydramine	10	2
Intervention	Epinephrine	0.3	30	Diphenhydramine	10	14
Intervention	Epinephrine	0.3	50	Diphenhydramine	10	15
Intervention	Epinephrine	0.1	49	Diphenhydramine	10	23
Intervention	Epinephrine	0.3	15	Diphenhydramine	10	2
Intervention	Epinephrine	0.1	2	Diphenhydramine	10	28
Control	Epinephrine	0.1	28	Diphenhydramine	10	19
Control	Epinephrine	1	56	Diphenhydramine	10	16
Control	Epinephrine	0.1	27	Diphenhydramine	22	35
Control	Epinephrine	0.2	15	Diphenhydramine	10	65
Control	Epinephrine	0.2	30	Diphenhydramine	1	51
Control	Epinephrine	0.1	25	Diphenhydramine	10	35
Control	Epinephrine	0.1	10	Diphenhydramine	10	46
Control	Epinephrine	0.2	21	Diphenhydramine	10	28
Control	Epinephrine	0.1	7	Diphenhydramine	12	43
Control	Epinephrine	0.1	10	Diphenhydramine	not given	not given

**Table 3a:** Dose error in each group.

	Number of dosing errors
Control group	
Acetaminophen	1
Midazolam	1
Epinephrine	1
Diphenhydramine	2
Intervention group	
Acetaminophen	0
Midazolam	0
Epinephrine	0
Diphenhydramine	0

**Table 3b:** Time-to-medication delivery in each group.

	Control group	Intervention group	p-value
Acetaminophen	50.6	28.6	0.036
Midazolam	27	8.6	0.005
Epinephrine	22.9	23.3	0.96
Diphenhydramine	37.6	13	<0.005
